# FREQ-Seq: A Rapid, Cost-Effective, Sequencing-Based Method to Determine Allele Frequencies Directly from Mixed Populations

**DOI:** 10.1371/journal.pone.0047959

**Published:** 2012-10-31

**Authors:** Lon M. Chubiz, Ming-Chun Lee, Nigel F. Delaney, Christopher J. Marx

**Affiliations:** 1 Department of Organismic and Evolutionary Biology, Harvard University, Cambridge, Massachusetts, United States of America; 2 Faculty of Arts and Sciences Center for Systems Biology, Harvard University, Cambridge, Massachusetts, United States of America; University College London, United Kingdom

## Abstract

Understanding evolutionary dynamics within microbial populations requires the ability to accurately follow allele frequencies through time. Here we present a rapid, cost-effective method (FREQ-Seq) that leverages Illumina next-generation sequencing for localized, quantitative allele frequency detection. Analogous to RNA-Seq, FREQ-Seq relies upon counts from the >10^5^ reads generated per locus per time-point to determine allele frequencies. Loci of interest are directly amplified from a mixed population via two rounds of PCR using inexpensive, user-designed oligonucleotides and a bar-coded bridging primer system that can be regenerated in-house. The resulting bar-coded PCR products contain the adapters needed for Illumina sequencing, eliminating further library preparation. We demonstrate the utility of FREQ-Seq by determining the order and dynamics of beneficial alleles that arose as a microbial population, founded with an engineered strain of *Methylobacterium*, evolved to grow on methanol. Quantifying allele frequencies with minimal bias down to 1% abundance allowed effective analysis of SNPs, small in-dels and insertions of transposable elements. Our data reveal large-scale clonal interference during the early stages of adaptation and illustrate the utility of FREQ-Seq as a cost-effective tool for tracking allele frequencies in populations.

## Introduction

The textbook definition of evolution is the change in frequency of alleles in a population over time [1]. While this extremely simple definition provides a clear picture of the evolutionary process, the ability to actually detect and track the frequency of known mutations in populations had previously been limited to work on phage [2], and remains surprisingly challenging for microbes. Identifying evolved alleles by applying next-generation sequencing technologies either to isolates [3], [4], [5] or mixed-population samples [6] has revolutionized the discovery of polymorphisms that occur during adaptation. However, this information does not often yield quantitative allele frequency information. The classic method for determining allele frequencies - examination of a large number of isolates from a population [7], [8], [9] - is extremely time- and resource-intensive. Several methods exist that allow accurate determination of allelic frequencies directly from population samples, but these either require substantial material and personnel cost and/or have limited sensitivity and accuracy [10], [11], [12]. For these reasons, a reliable, high-throughput pipeline for allele frequency detection in natural and laboratory populations will allow a more extensive understanding of evolutionary processes.

Considering the equipment present in most molecular biological laboratories, there exist only a handful of techniques available to researchers to determine the alleles present in isolates, or their population frequencies directly from mixed samples. The simplest and most time-honored of these methods is restriction fragment-length polymorphism (RFLP) analysis. Although generally quite accurate, this technique requires the polymorphisms under investigation to change restriction endonuclease cleavage sites or grossly change the sequence length [13], [14]. This limits application to a small subset of mutations. Additionally, a number of quantitative PCR-based (qPCR) methods have shown promise though their implementation requires significant optimization and calibration [3], [15]. Lastly, and arguably the most straight-forward method, has been the use of Sanger sequencing to determine allelic frequencies in mixed DNA samples [3], [9], [16]. The chief drawbacks of this method are that accuracy and detection limits are highly variable requiring a large number of replicate samples.

In recent years, a number of commercial systems have been developed to quantitate allele frequencies [17], [18], [19], [20], [21]. The Sequenom iPLEX® platform utilizes differential PCR extension coupled with matrix assisted laser desorption/ionization – time of flight (MALDI-TOF) mass spectrometry to infer relative allele frequencies in a given pooled sample. Alternatively, the Fluidigm Access Array™ and Raindance Thunderstorm™ systems use proprietary, dedicated microfluidic thermal cyclers for targeted PCR enrichment of variable loci followed by deep-sequencing using any number of next-generation sequencing platforms. Additionally, use of the Access Array™ and Thunderstorm™ systems require synthesis and re-ordering of pairs of individual, long oligonucleotides for each allele probed. Although these methods have been successfully applied, the substantial equipment and/or supplies costs pose barriers to most academic end-users.

**Figure 1:**
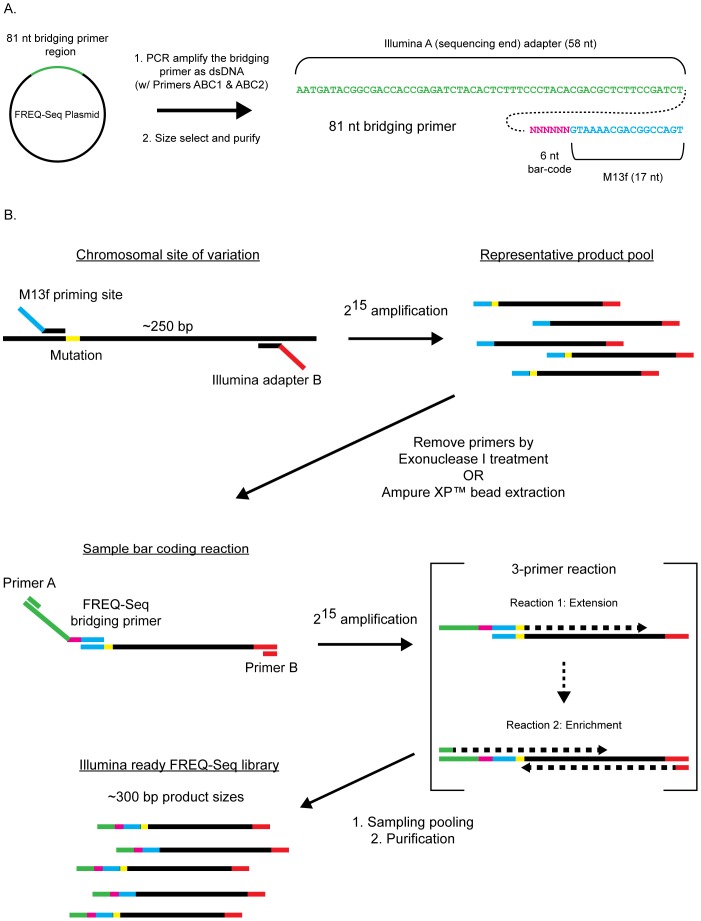
Illustration of the FREQ-Seq method. A.) The bridging primers can be produced in house using standard PCR and purification. Individual, uniquely bar-coded bridging primers are amplified from the FREQ-Seq plasmid library set using two primers ABC1 (AATGATACGGCGACCAC) and ABC2 (ACTGGCCGTCGTTTTAC). The resulting bridging primers are stored as dsDNA at −20°C. B.) Construction of FREQ-Seq Illumina libraries is conducted in two stages. A first round of PCR amplification is used to generate an allele-specific, representative fragment pool with product inserts approximately 250 bp in size. Each product at this stage contains an M13f sequence at the sequencing end and an Illumina B adapter sequence at the non-sequencing end. These products are then treated with exonuclease I or Ampure XP beads to remove remaining PCR primers. In a second stage of PCR amplification, individual samples are barcoded and enriched using three primers; a small amount of a FREQ-Seq bridging primer and two Illumina enrichment primers. Initially, amplification proceeds via the sample-specific bridging primer that has the M13f sequence at its 3′ end. The resulting products can then be amplified to higher quantity with the generic, enrichment primers A and B. Following sample pooling and standard PCR product purification, the resulting products constitute a complete FREQ-Seq library.

In this work, we have developed a rapid, cost-effective strategy for localized, Illumina-based allele “frequency sequencing”, or “FREQ-Seq”. The basic rationale of FREQ-Seq is analogous to RNA-Seq: allele frequencies (rather than transcript levels) are determined by counting DNA sequence reads. FREQ-Seq utilizes two rounds of PCR that take advantage of the inclusion of a small amount of a long, bridging primer that can be produced and re-generated in-house at low cost in order to introduce sample-specific bar-codes (**Figure 1**). Similar to Access Array™ and Thunderstorm™, this method applies PCR enrichment and next-generation sequencing platforms to estimate allele frequencies, but in our case, without the expense of individual long oligonucleotides for each locus, nor the need for additional instrumentation. The resulting PCR products require no further library preparation for sequencing, and are simply pooled together for analysis using either Illumina HiSeq or Genome Analyzer platforms. The resulting libraries, introduced at just 5% of an Illumina HiSeq control lane, produced an average of 147,000 reads per sample. With a custom, publicly available analysis software package we have developed, users can easily extract and interpret experimental results.

We validated and demonstrated the utility of the FREQ-Seq system by determining the frequency of four beneficial mutations that arose during adaptation of an engineered *Methylobacterium extorquens* AM1 strain that requires a heterologous pathway for formaldehyde oxidation [22]. We examined both i) controlled cellular ratios of each allele and ii) cryogenically stored population time-point samples. This engineered strain had been evolved for 900 generations in the laboratory in a minimal medium containing methanol as a sole carbon source [23]. Use of these allele frequency data allowed for direct comparison of observed fitness increases to the rapid rise and selective sweep of these mutations. These data revealed a period of clonal interference that temporarily reversed the rise of alleles that would later move to fixation. For simple polymorphisms, like SNPs and small in-dels we found calibration was unnecessary, whereas analysis of more complex allele types such as >10 bp deletions benefit from correction via control ratios and a simple model fit. Finally, we demonstrate that a modified primer strategy allows FREQ-Seq to be used to analyze new DNA junctions such as novel transposable element insertions which have been repeatedly observed in this [24] and other evolution experiments [13], [14].

## Materials and Methods

### Strains, culturing and automated growth rate measurements

All strains used are derivatives of *Methylobacterium extorquens* AM1 with relevant genotypes listed in **Table S4**. Bacterial cultures were routinely grown in ‘Hypho’ minimal medium exactly as described by Chou et al. [24], supplemented with 3.5 mM succinate or 15 mM methanol, unless otherwise described, and was performed at 30°C with aeration. For growth rate measurements, 5 µL frozen population cultures were inoculated in 640 µL medium supplemented with 13.125 mM methanol and 0.4375 mM succinate (ratio of carbon from M∶S = 7∶1) in 48-well micotiter plates (Costar) and grown to saturation to acclimate to growth on methanol. Following acclimation, saturated cultures were transferred with a 1∶64 dilution in fresh Hypho medium with 20 mM methanol and placed on a plate shaking tower (Caliper) at 30°C in a humidified environmental room. Optical densities were obtained every 2 hrs on a Wallac Victor 2 plate reader (Perkin-Elmer) until cultures reached saturation using an automated measurement system [25]. Growth rates were determined by fitting an exponential growth model using custom analysis software, Curve Fitter (Delaney et al., unpublished; http://www.evolvedmicrobe.com/Software.html), with a minimum of 4 replicates.

### Construction of a bar-coded adapter plasmid library

The entirety of the adapter library was assembled in a vector backbone for ease of storage and routine amplification. To generate an appropriate plasmid backbone, the pUC19 vector (New England Biolabs) was PCR amplified using primers TCGGTGGTCGCCGTATCATTTTAATTGCGTTGCGCTCACTG and AGAGTAAAACGACGGCCAGTTACGCATCTGTGCGGTATTTC, and Phusion DNA polymerase (New England Biolabs, NEB) under manufacturer recommended conditions, to generate a linear DNA fragment containing only the replication origin and ampicillin resistance marker. The absence of the *lacZα* multiple cloning site ensures no interference with downstream PCR amplification using the M13f priming site. The resulting PCR reaction was treated with 20 units of *Dpn*I restriction endonuclease (NEB) for 1 hr at 37°C to remove the pUC19 plasmid template prior to purification of the linear DNA product. The bridging primer was first synthesized and PAGE purified (Integrated DNA Technologies, IDT) using the template AATGATACGGCGACCACCGAGATCTACACTCTTTCCCTACACGACGCTCTTCCGATCTNNNNNNGTAAAACGACGGCCAGT, where N signifies random nucleotides comprising the unique bar code. The subsequent 81 nt ssDNA oligonucleotide was then converted into dsDNA by five cycles of PCR using the primer ACTGGCCGTCGTTTTAC and Phusion DNA polymerase under standard reaction conditions with a 5 s extension time. Presence of dsDNA following PCR was verified by 10% Acylamide/1X TAE electrophoresis and SybrSafe staining. The resulting DNA was then purified using phenol extraction and ethanol precipitation.

To create the plasmid-borne bar-code library, 100 ng of the linear pUC19 PCR product as well as an equimolar amount of the 81 bp dsDNA Illumina-M13f bar-code were mixed and assembled into circular plasmid DNA using the one-step method developed by Gibson and co-workers [26]. Approximately 20 ng of the resulting assembled DNA was then transformed into NEB10β cells using the 5 minute transformation protocol (NEB) to eliminate sibling clones, followed by plating of 100 µl aliquots onto SOB plates containing ampicillin (100 µg/mL) and X-Gal (80 µg/mL). The resulting white, single colonies were inoculated into 1 mL of SOB with ampicillin (100 µg/mL), grown overnight at 37°C in deep, 96-well plates with shaking at 320 rpm, and stored at −80°C after the addition of DMSO to 8% final concentration. Approximately, 192 clones were verified by Sanger sequencing (MWG Operon) and a resulting set of 48 bar-coded adapters were picked based on a minimum Hamming distance of 2.

The entire FREQ-Seq barcode library is publicly available through Addgene.org and available individually or as a full kit of 48 plasmids (www.addgene.org/Christopher_Marx/).

### Amplication and purification of the bar-coded Illumina-M13f bridging primers

Production of dsDNA Illumina-M13f bridging primers for sample bar-coding was performed by PCR followed by agarose gel extraction. To amplify a given adapter from the plasmid adapter clone library, PCR reactions were formulated with primers ABC1 and ABC2 (**Table S1**) using Phusion DNA polymerase and cycled under standard conditions with a 5 s extension time. The resulting products were purified by 2% agarose gel electrophoresis and gel extraction (Zymo ZR-96 kit) according to manufacturer's instruction. Illumina-M13f bridging primers were then verified for correct size and purity using an Agilent Bioanalyzer.

### Allele frequency estimates as determined by flow cytometry

Allele frequency mixtures were generated by growing each isogenic allele type found in **Table S4** to late-log phase. Subsequent mixtures were made using cell cultures diluted to OD_600_ = 0.15. Ratios of cell types were measured using a BD LSRII flow cytometer. Estimates of allele frequency were generated using the FlowCore BioConductor package in R to gate fluorescent and non-fluorescent cell types [27].

### Allele specific library generation, sequencing and frequency determination

Allele-specific, Illumina-compatible FREQ-Seq sequencing libraries are generated via a two-step procedure outlined in **Figure 1B**. A basic overview of the protocol is provided in **Box S1**. Additional workflow examples may be found in the online **Text S1**. In the initial step, the region containing genetic variation (SNPs or deletions) is amplified by PCR using allele-specific primers containing overhangs shown in **Figure 1B** and listed in **Table S1**. These PCR products thereby contain the region of interest flanked by the non-sequencing Illumina adapter sequence on one end (Illumina “B”) and a ‘universal adapter’ (M13f sequence) on the sequencing end to enable subsequent bar-coding. Notably, any polymorphisms must be positioned closely (e.g., <5–10 bp for 50-bp reads; 55–60 bp for 100-bp reads) to the end with the universal adapter in order to enable detection. This is due to the first 40–45 bases of a sequencing read being consumed by the FREQ-Seq related barcodes and adapters (**Figure 1A**). Thus, for each new locus, only two primers need to be synthesized as standard DNA oligonucleotides. The second round of PCR is unique in that it uses three primers. The outer primers are generic to all reactions and utilize the Illumina “A” and “B” sequences to amplify all products. Critical to our method, a small amount (1/10 normal concentration) of a long, bridging primer is doped into each reaction. These 81-mers have the Illumina “A” sequence at the 5′-end, the M13f adapter sequence at the 3′-end to amplify the templates from the first round of PCR, and a 6-mer bar-code in between that allows each amplicon pool to be identified in a sample-specific manner (see **Figure 1** and **Tables S1** and **S2**). This results in production of DNA fragment pools already compatible with the Illumina single-end read flow cell, obviating all subsequent library preparation steps (e.g., shearing, adapter ligation, column selection, etc.). The resulting products are then pooled and sequenced *en masse* on a Illumina HiSeq or Genome Analyzer flow cell.

Following Illumina sequencing, data analysis is performed using custom, open-source software (FREQout), available at http://www.evolvedmicrobe.com/FreqSeq/index.html. The software includes a command-line executable and a program with a graphical user interface. Both programs are designed to read and align sequences contained in the Illumina FASTQ output file. User defined alleles and bar-code sequences, as well as alignment and other analysis parameters, are inputted to the software via an XML formatted file. The software runs the analysis by assigning each read to an allele and barcode group based on its sequence and the quality of its alignment to the different possible alleles (see also **Text S1**). Because the software checks for the presence of the M13f sequence while parsing the reads, this step allows for FREQ-Seq samples to be run in parallel with other samples on the same Illumina flow cell. The data generated on the frequency of each type, as well as statistics related to various quality and alignment metrics, are then written to a CSV file. The graphical user interface also includes interactive plots that allow users to view these details immediately.

## Results

### Method validation

In order to determine the efficacy of FREQ-Seq, we sought to address three primary concerns: the scalability of library preparation, sources of bias, accuracy in the measurements, and the detection limits of the technique. To ensure scalability, we designed our ‘bridging’ primer system, that introduces the sample-specific barcode, in a manner that can be produced in-house (**Figure 1A** and **Text S1**). This alleviates the significant cost and high error rates of custom synthesis of >60 bp DNA oligonucleotides. Specifically, we found that approximately 15% of clones from the FREQ-Seq bridging primer plasmid library contained frame-shift or mismatch mutations, apparently introduced during oligonucleotide synthesis, and were thus culled. Presently, we have experimentally confirmed 48 unique bar-coded adapters all possessing sequence Hamming distances greater than two. This criterion allows for greater fidelity in bar code assignment during data processing. In addition, these 48 bar codes, positioned to be the very first bases sequenced, contain no significant nucleotide bias within the first four bases. This is critical because biases in the first four bases of Illumina reads are known to cause significant reductions in cluster identification resulting in large numbers of abandoned reads by the Illumina analysis software [28].

**Figure 2:**
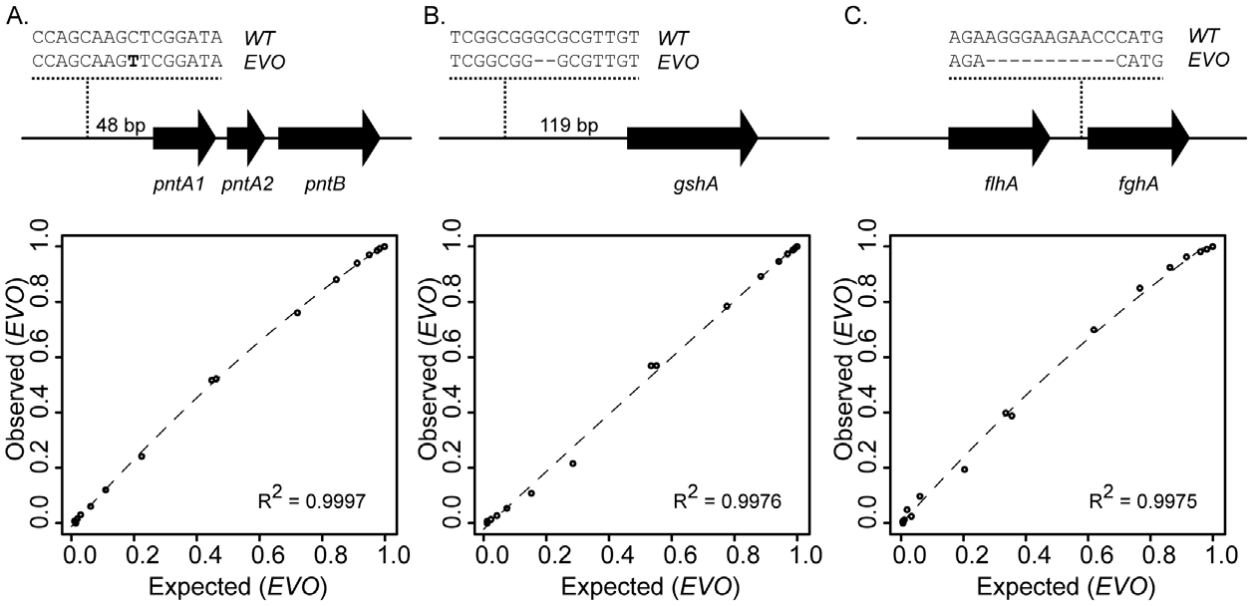
Calibration of FREQ-Seq data for three ancestral/evolved (WT/EVO) allele mixtures *pntAB^EVO^* (A.), *gshA^EVO^* (B.), and *fghA^EVO^* (C.). Strains bearing each evolved allele were compared to one with the wild-type version that also expressed mCherry to permit precise quantification of expected ratios by flow cytometry (**Table S3**). Presented in each column are sequence variation between the ancestral WT and EVO alleles, and subsequent quadratic fits of the data for each respective allele. Observed data are the frequencies of the evolved allele as determined by FREQ-Seq, whereas expected data are wild-type allele frequencies estimated using flow cytometry. Quadratic model fitting was performed in R.

Since sequencing libraries require PCR amplification, we examined the potential bias that could arise from differential product amplification or detection. As controls, we assayed mixtures containing a wide range of cell ratios – accurately determined via flow cytometry – containing either the ancestral or evolved version of an allele. Following 600 generations of adaptation to methanol growth by a genetically engineered strain of *M. extorquens* AM1, an isolate was chosen and sequenced to determine the genetic basis of adaptation [22]. We began our analysis of this population by interrogating three beneficial mutations, *pntAB^EVO^*, *gshA^EVO^*, and *fghA^EVO^*, which span the gamut of a SNP and deletions of 2- and 11-bp, respectively (**Figure 2A–C**).

Comparison of the observed allele frequencies compared to those expected from the cell ratios demonstrated that there was remarkably little bias throughout the procedure (**Figure 2**). Only the 11-bp deletion of the *fghA* polymorphism deviated to somewhat from expectation (**Figure 2C**). . Because, fortuitously, each of the allele types of *pntAB*, *gshA*, and *fghA* resulted in change in restriction pattern, we used quantitative RFLP analysis of the same amplicon pools and found a similarly weak bias (**Figure S1**). Fitting these control data to a quadratic function, however, allowed us to readily control for the minor bias of *fghA*, but it appears to be largely unnecessary for simple SNPs or small in-dels.

The FREQ-Seq method can consistently produce allele frequency estimates with low absolute errors. However, for very high and very low allele frequency estimates (>99% or <1%), the relative error on the estimates can become large because the frequency of the allele (the signal) starts to become equal to or less than the frequency of sequencing errors (the noise). In principle, one could obtain better estimates of very low or high frequency alleles by making the estimate within a probabilistic framework that accounts for the chance that a read is a sequencing error. This would require a model for the likelihood of sequencing errors, which would be best verified by running a sample that is entirely of one type and seeing how often the other type appears. We performed this assay by sequencing barcoded samples for both the *gshA* and the *pntAB* loci that were at 100% frequency for only one allele type, and did this with four independently prepared samples for both alleles on four different dates. For both alleles, and for all sequencing dates, the observed error rate was very low and the median observed percentage of the type not supposed to be present in the sample was 0.23%, indicating that this percentage is approximately equal to the range in which signal and noise become equally important to the direct allele frequency estimate. However, interestingly, a comparison of the different dates shows that the frequencies of incorrect sequencing reads in the samples were statistically different for both allele types (logistic regression, p-values<10^−15^), and two samples from one date contained errors that were greater than 1% at 1.41% and 1.13% respectively. We interpreted this result to mean that the frequency of sequencing errors can be influenced by subtle and distinct biases for different sequencing runs, and so one must be careful before applying an error model trained on one day's data to sequencing data obtained on another day. However, once data from many more sequencing runs is obtained, we suspect one could create a reliable method to more accurately assess uncertainty in low and high frequency samples by using a hierarchical model that accounts for day to day variation and that also includes read quality scores as a covariate.

### FREQ-Seq can be used to monitor novel DNA junctions

**Figure 3:**
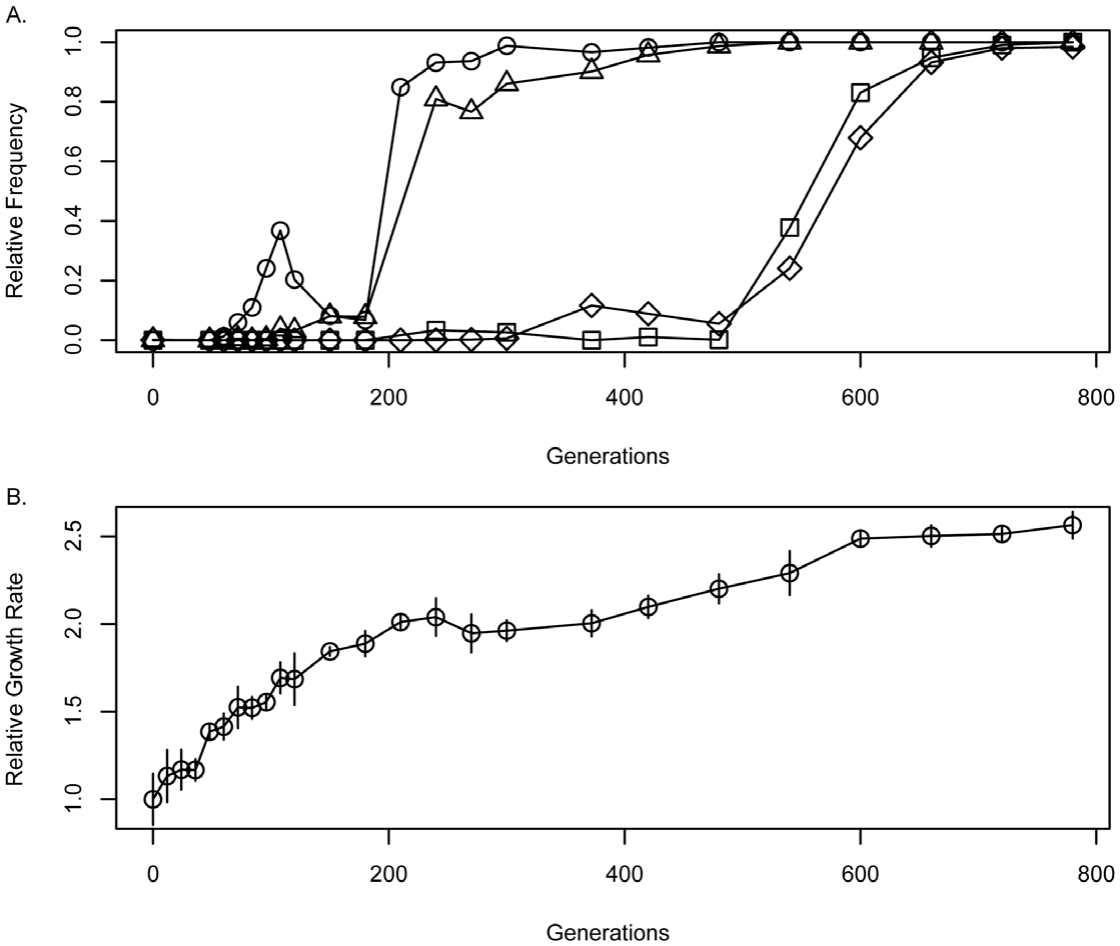
Allele frequencies for four beneficial alleles in population F4 (A) with correlated changes in relative growth rate (B). Alleles *gshA^EVO^* (circles), *fghA^EVO^* (triangles), *pntAB^EVO^* (squares), and *icuAB^EVO^* (diamonds) were determined by FREQ-Seq and corresponding values were corrected with calibrated data shown in **Figure 2**. Growth rates (B.) were normalized to the growth rate of the ancestral strain.

A reoccurring mutational type in many evolution experiments is the formation of new DNA junctions, either by deletion, amplification or insertion sequence (IS) element/transposon insertions [4], [13], [14], [23], [24], [29], [30], [31]. To assess the ability of FREQ-Seq to detect new DNA junctions we modified our primer strategy to use three primers in the first reaction (two forward, sequencing-end and one reverse found in **Table S1**, see **Figure S2**) to amplify a region adjacent to the *icuAB* locus on the *M. extorquens* AM1 chromosome. This site was previously shown to contain an IS element insertion that resulted in the increased expression of *icuAB* (a cobalt transport system) under metal limitation [24]. Of the three primers designed, the two forward, sequencing-end primers were designed to differentially bind to the *icuAB^WT^* and *icuAB^EVO^* (IS containing) genotypes, while the third reverse primer annealed to a common site downstream of the IS insertion site. Thus, each amplicon would only occur in the presence of *icuAB^WT^* or *icuAB^EVO^*, respectively. Using the same protocol developed for two locus primers, we found that FREQ-Seq was capable of detecting the IS element insertion located proximal to *icuAB* and allowed us to follow its rise to fixation (**Figure 3**).

### Determining the order and dynamics of beneficial alleles from evolved populations

Numerous evolution experiments have been performed using microbial populations where temporal population samples have been cryogenically stored. Wide-spread access to whole genome resequencing has allowed many of the beneficial mutations that have risen in such populations to be identified and characterized. For the *M. extorquens* AM1 population we have used as a test case, we had previously uncovered a generic pattern of epistasis between the mutations that emerged that led to diminishing returns [23]. What was unknown, however, was the order and dynamics by which these beneficial alleles emerged. Therefore, to elucidate the adaptive trajectory and dynamics for this population we employed FREQ-Seq to the three loci described above (**Figure 2**), as well as *icuAB*. Interestingly, we discovered that the order of incorporation of these alleles followed the ranking of their selective advantages: *gshA^EVO^*, *fghA^EVO^*, *icuAB^EVO^* and then *pntA^EVO^* (**Figure 3**). Despite the large selective advantage of the alleles we examined, we uncovered a substantial, temporary reversal in relative frequencies between generations 108 and 210. This pattern is indicative of clonal interference (i.e. the Hill-Robertson effect; [32]); whereby multiple lineages accrue beneficial mutations and interfere with each other's path toward fixation [31], [33], [34], [35], [36].

### Comparison of FREQ-Seq data to genotype data directly from random isolates

**Figure 4:**
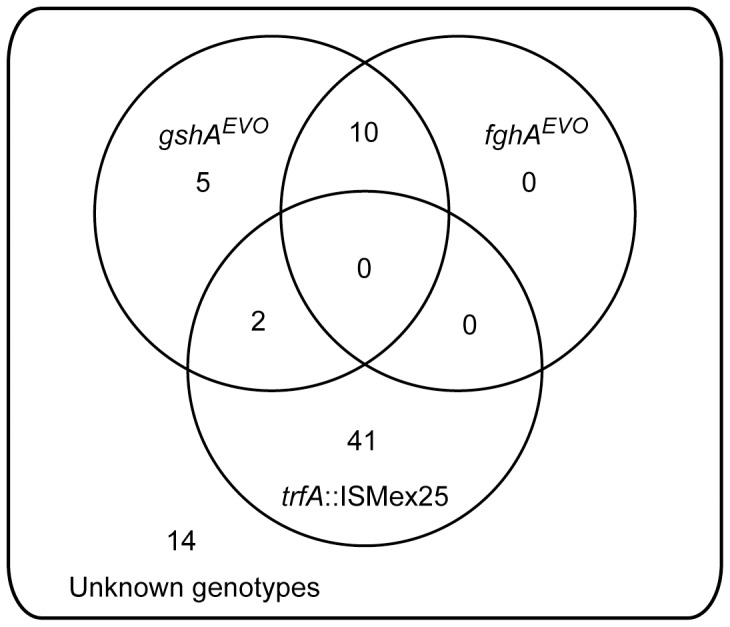
Genotyping of 72 isolates from population F4 at generation 150. Genotypes were determined by PCR amplification and RFLP analysis using enzymes *Hha*I and *Hpy*AV for the *gshA*, and *fghA* alleles, respectively. *trfA*::ISMex25 alleles were assayed by standard PCR amplification.

The FREQ-Seq data from our test population suggested three things: the order of mutations, probable early linkage of *gshA^EVO^* and *fghA^EVO^* before they both dramatically rose in frequency, and that the majority of the population at generation 150 had unidentified mutations. As an independent method to test these inferences, we obtained and independently genotyped 72 random isolates from generation 150 (**Figure 4** and **Table S2**). First considering the known alleles from the eventual winning lineage that were present at this time (*gshA^EVO^* and *fghA^EVO^*), isolates were found with both *gshA^EVO^* and *fghA^EVO^*, or just *gshA^EVO^*, but not just *fghA^EVO^*. This pattern and linkage data is consistent with the FREQ-Seq inferences that the *fghA^EVO^* mutation arose later and on the background of *gshA^EVO^*.

Turning to trying to uncover what other mutations were present in the lineages that temporarily outcompeted those with *gshA^EVO^* and *fghA^EVO^*, we considered previous work on parallelism across the replicate populations [27]. This paper had found that all populations contain mutations that, like the *fghA^EVO^* allele described here, reduce expression of the introduced formaldehyde oxidation pathway in this strain. The most common mutational event across populations was IS-mediated co-integration of the introduced, multi-copy plasmid expressing the foreign formaldehyde pathway with an endogenous, single copy plasmid in *M. extorquens* AM1 [29]. We used PCR amplifications to determine whether such an event occurred in these isolates (**Table S3**). Of the 72 isolates, 43 of these had an *trfA*::ISMex25 allele (*trfA* encodes the plasmid replication protein of this IncP replicon, [37]). Interestingly, of these there were 2 that possessed both *gshA^EVO^* and an *trfA*::ISMex25 allele. This homoplasy is most likely due to repeated cointegrations on different backgrounds. Finally, there were 14 isolates that lacked all of the known beneficial alleles, but co-existed with the other genotypes at generation 150 when the growth rate of the population had risen by 84.4±2.5%. Clearly there were other, as yet unknown mutations that occurred and temporarily rose in frequency.

### Comparison of beneficial allele emergence with observed increases in population fitness

With a quantitative picture of genotypic change at key loci available via FREQ-Seq, we measured the change in growth rate of the population to determine if this corroborated the finding of clonal interference. We used growth rate as a proxy for fitness because it has been previously demonstrated for these populations that these two measures are linearly correlated [23]. As has been seen for other experiments with *M. extorquens* AM1 [38], as well as other model systems (e.g., [39]), the rate of fitness increase was faster early in the experiment then later, but continued to rise throughout. Importantly, the relative smoothness, rather than stepwise nature of the observed dynamics was consistent with theoretical models of clonal interference (and recurring “multiple mutations”; [40]). Furthermore, clear evidence for other lineages that were “invisible” to our FREQ-Seq data comes from the fact that the temporary peak of the *gshA^EVO^* allele at 34.7% of the populations occurred at generation 108, when the relative growth had already increased by 69.3±8.9%. Given that this was the inflection point between rising and falling in frequency, this indicates that the genotype containing *gshA^EVO^* had a fitness of ∼70%. This means that the rest of the population was somewhat less fit than this genotype prior to that time-point, and somewhat higher afterwards. Despite these fast reversals in allele frequencies during this interval, growth rate continued to rise relatively continuously. Compared to the wavering trajectory for *gshA^EVO^*, the trajectories of *fghA^EVO^*, and later *pntAB^EVO^* and *icuAB^EVO^* did not exhibit wild reversals, yet were more complex than the simple sinusoidal pattern expected were there to be periodic selection of single mutations in the absence of clonal interference [41].

## Discussion

Reported here is a method for quantitative determination of allele frequencies called FREQ-Seq. FREQ-Seq utilizes standard PCR and depends upon a novel bar-coding system to generate locus-specific Illumina sequencing libraries. This capitalizes on focusing the sequencing depth of next-generation sequencing platforms upon just the key loci of interest across timepoints or populations in order to accurately infer their frequency. A key aspect of FREQ-Seq is its capacity for multiplexing a large number of samples. For example, for academic laboratories engaged in experimental evolution or environmental microbiology, FREQ-Seq provides a low-cost, highly extendable method for investigating genetic variation over large temporal and spatial scales.

As a proof of principle we have demonstrated the utility of FREQ-Seq in tracking the emergence of four beneficial alleles from a previously characterized evolution experiment [23]. We also observed that, while the order of allele emergence matched the order which would have been the most optimal adaptive trajectory amongst the epistatic combinations generated by Chou and coworkers [23], a previously unknown period of clonal interference existed early during adaptation. Based on genotyping of clones during this period, one prominent source of interfering lineages resulted from IS insertions into the replication machinery (*trfA*) of the RK2-based plasmid harboring the exogenous formaldehyde oxidation pathway. Similar IS insertions have recently been found in many of the replicate populations from this experiment, and provide 17.4–24.1% fitness increases due to reducing the cost associated with exogenous pathway expression [29]. Interestingly, while these IS mutational variants were the eventual evolutionary ‘winners’ in other populations, they were unable to outcompete the *fghA^EVO^ gshA^EVO^* genotype despite being simultaneously present in *gshA^EVO^* backgrounds. These results underpin the importance of allele frequency data in revealing the complex processes during adaptation. Furthermore, by simultaneously determining the population growth rate with high resolution, we were able to both estimate the fitness of the genotype containing the allele of interest and demonstrate that the reversal in its frequency actually occurred during a period of continued rise in fitness. As more studies move to characterizing improvement accurately and with fine time resolution, the synergistic role of FREQ-Seq estimates of allele frequencies will become even more important to connect between phenotypic and genotypic change.

This test case highlights advantages of the FREQ-Seq method. First, our results show that a wide range of allelic types can be detected ranging from SNPs and small in-dels to novel IS junctions. Second, operating at approximately 5% of an Illumina flow cell capacity we were able to achieve >10^5^ reads per locus per time-point. Third, we observed minimal bias in our control ratios. This suggests that FREQ-Seq is fully capable of quantitatively detecting these types of alleles with little or no calibration required, particularly for SNPs or small in-dels. Where needed a simple quadratic fit can be used to account for small, systematic bias. We encourage potential users to consider a control mixture to account for possible skews in observed frequencies for these types of alleles. Fourth, the sensitivity available is quite reasonable. Our data allowed us to quantify allele frequencies reliably from 1–99%. More sophisticated statistical error models or increased flow cell capacity can likely increase the sensitivity of FREQ-Seq. Fifth, the publicly available FREQout analysis package allows for researchers to easily recover data and receive a full report of allele frequency types.

A critical component of any new method is the cost of implementation, and the associated tradeoff in accuracy and throughput, relative to existing techniques and technologies. With this in mind, we explored the cost of FREQ-Seq implementation compared to existing high-throughput platforms (Fluidigm AccessArray™ and Raindance Thunderstorm™) and sequencing-based allele frequency detection methods (Sanger sequencing and whole genome resquencing). As an initial cost comparison, we chose to examine two commercial systems from Fluidigm and Raindance that use a very similar strategy for PCR enrichment and barcoding of chromosomal loci. FREQ-Seq and these two platforms both require the use of locus specific oligonucleotide primers containing long overhangs. Unlike FREQ-Seq, however, both the Fluidigm and Raindance systems require long barcoded primers, containing both Illumina adapter sequences, for every locus of interest. While an unassuming difference, the latter primers routinely exceed most standard oligonucleotide synthesis lengths (currently 60 nucleotides at $0.35 US/base, IDT) resulting in increased synthesis and purification costs (>$1 US/base). Additionally, as these primers are locus specific, each locus must have a new set of barcoded primers. To directly compare, each Fluidigm or Raindance barcoded primer set would cost approximately $3,120 US (48×65 nt barcoded primers at approximately $1 US/base) for a single locus whereas a FREQ-Seq locus primer set of similar size costs $32 US (1×37 nt primer +1×54 nt primer at $0.35 US/base) as all barcoding is accomplished with the universal bridging primer in a locus-indendent manner. Finally, comparing equipment costs (assuming equal amounts of PCR reagents and user time are used) shows that the aforementioned commercial technologies (not considering annual custom consumables and instrument servicing costs) range between $70,000 to $100,000 US. In contrast, FREQ-Seq relies only on a standard, 96-well PCR thermal cycler already present in or generally available to most laboratories. Collectively, FREQ-Seq is two orders of magnitude less expensive in oligonucleotide costs and again obviates purchasing an expensive equipment specifically designed for this purpose. Further, as FREQ-Seq is an open-source platform, no reagents or equipment are proprietary, thereby alleviating significant annual service and licensing costs for small research laboratories and institutions.

Additionally, one may consider alternative strategies such as traditional Sanger sequencing and whole genome resequencing to infer allele frequencies in mixed populations. Using the analysis of an experimentally evolved population demonstrated in **Figure 3** as a metric, Sanger sequencing of four loci for 22 time points would result in a cost for a single set of replicates to be $440 US (5 $US/reaction, GeneWiz). However, to achieve any statistical accuracy, multiple replicate samples for each allele and time point would need to be run. Moreover, the accuracy generated by Sanger sequencing is routinely difficult to achieve below 10% allele abundance [3], [9], [16]. Likewise, employing the same logic to whole genome resequencing of mixed population samples using the Illumina HiSeq platform, approximately 12 samples can be run per lane to produce an average coverage of 100–200 fold. Conservatively, this experiment would result in the use of two full lanes in an Illumina flow cell (costing approximately $2,400 US using Harvard FAS Core pricing) and would require 22 barcoded Illumina libraries to be generated (an additional cost of $1,200 US with TruSeq preparations at $55 US/sample). An immediate benefit of this strategy is that nearly all chromosomal mutations can be seen simultaneously. The drawback is that 100–200 fold coverage results in both substantial sampling error in estimating alleles of intermediate frequency, and is insufficient to detect alleles at low frequency, resulting in increased coverage being necessary (with costs increasing linearly). In contrast, using only ∼3% of a single Illumina flow cell lane ($40 US), FREQ-Seq is able to achieve an average of 100,000 fold coverage per allele, per time point. If we thus consider the cost per 1,000 fold coverage, whole genome sequencing would cost ∼$700 US per time point, whereas FREQ-Seq costs $0.005 US per locus of interest in the population. By focusing on loci of interest, FREQ-Seq uses the five order of magnitude difference in cost per coverage to achieve robust quantitative results and sub 1% frequency detection. Taken together, these comparisons demonstrate that FREQ-Seq is a cost-effective, quantitative strategy for localized allele frequency determination.

In the present implementation of FREQ-Seq, we have described a set of 48 uniquely bar-coded bridging primers to produce localized Illumina sequencing libraries compatible with either single-end or paired-end read flow cells. Although currently designed for single-end reads, changing compatibility to paired-end flow cells would only require simple modification to the reverse locus primer to change its 5′ overhang, and an identical change in the primer ‘B’ for enrichment (**Table S1**). Though employed here to explore the frequencies of known alleles in a laboratory evolution experiment, we expect FREQ-Seq to be useful in other diagnostic applications. Multi-way competitions between bar-coded strains could be readily performed. Moving out of the lab entirely, microbial species variation in environmental samples is entirely feasible. For example, phylogenetic analyses of 16S rRNA from communities could occur with excellent throughput and no subsequent library preparation. As a result, FREQ-Seq should be a useful tool to many biomedical and environmental microbiologists.

## Supporting Information

Figure S1 **Estimation of PCR-introduced bias in allele amplification.** Allele *pntAB^EVO^* (circles), *gshA^EVO^* (squares), and *fghA^EVO^* (triangles) were amplified using primer pairs AF3/AF4, AF7/8, and AF9/10, respectively followed by bridging primer (MLBC1) addition. Final FREQ-Seq products for *pntAB^EVO^*, *gshA^EVO^*, and *fghA^EVO^* were digested with *Hha*I, *Alu*I, or *Hpy*AV, respectively. Digested DNA fragments were sized fractionated and analyzed using a Bioanalyzer 2100 (Agilent) with DNA1000 chips. Ratios were determined using the relative ratios of integrated peak areas from the Bioanalyzer trace. Data were analyzed using the Agilent 2100 Expert software package.
(TIF)

Figure S2 **Schematic of the three primer, novel DNA junction implementation of FREQ-Seq.**
(TIF)

Box S1 **Abbreviated FREQ-Seq protocol.**
(TIF)

Table S1 **FREQ-Seq primer sequences and allele-specific primers used in this study.**
(DOCX)

Table S2 **Sequences of the 48 barcodes contained in the FREQ-Seq kit.** All sequences are the N bases in the sequence AATGATACGGCGACCACCGAGATCTACACTCTTTCCCTACACGACGCTCTTCCGATCT**NNNNNN**GTAAAACGACGGCCAGT.
(DOCX)

Table S3 **Genotypes of 72 isolates from population F4 at generation 150.** An (X) indicates the presence of an evolved allele (EVO) as determined by RFLP or differential PCR amplication.
(DOCX)

Table S4 **Bacterial strains used in this study and their relevant genotypes.**
(DOCX)

Text S1 (DOCX)
