# Supplementary figures and images for: FREQ-Seq: A Rapid, Cost-Effective, Sequencing-Based Method to Determine Allele Frequencies Directly from Mixed Populations
Source: PLoS One. 2012 Oct 31;7(10):e47959. doi: 10.1371/journal.pone.0047959 (PMC3485326; doi:10.1371/journal.pone.0047959)

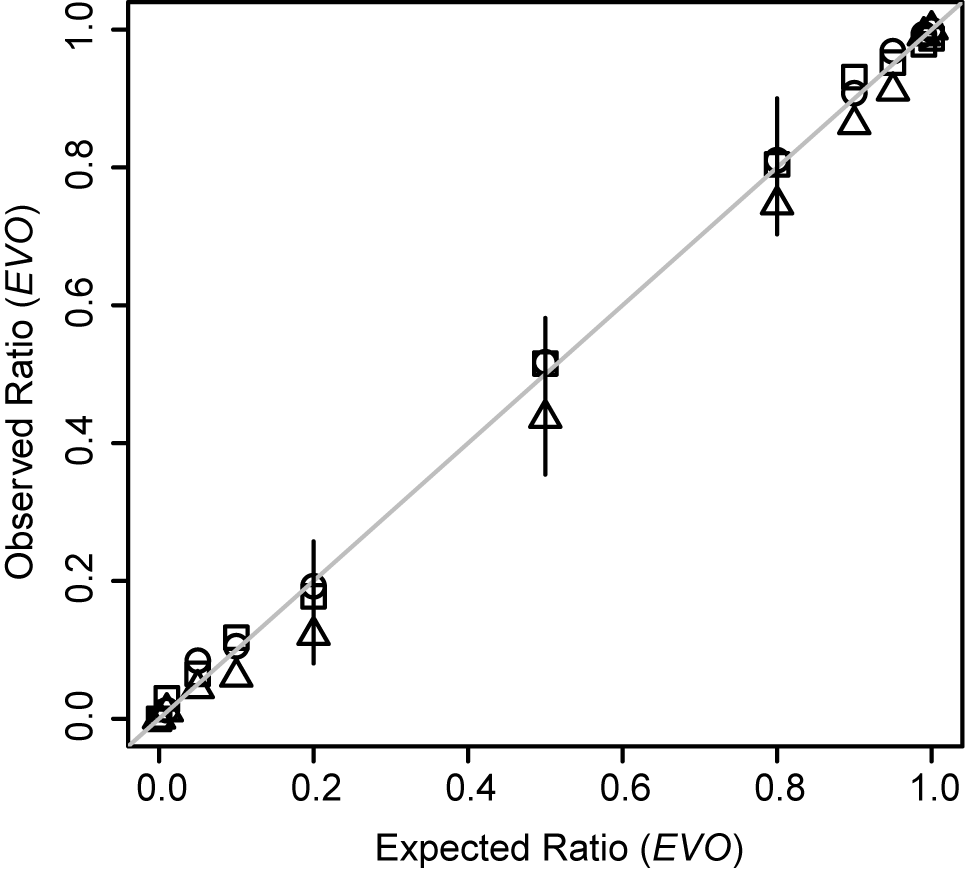

Supplement: Figure S1 — Estimation of PCR-introduced bias in allele amplification. Allele pntABEVO (circles), gshAEVO (squares), and fghAEVO (triangles) were amplified using primer pairs AF3/AF4, AF7/8, and AF9/10, respectively followed by bridging primer (MLBC1) addition. Final FREQ-Seq products for pntABEVO, gshAEVO, and fghAEVO were digested with HhaI, AluI, or HpyAV, respectively. Digested DNA fragments were sized fractionated and analyzed using a Bioanalyzer 2100 (Agilent) with DNA1000 chips. Ratios were determined using the relative ratios of integrated peak areas from the Bioanalyzer trace. Data were analyzed using the Agilent 2100 Expert software package. (TIF) [file pone.0047959.s001.tif]

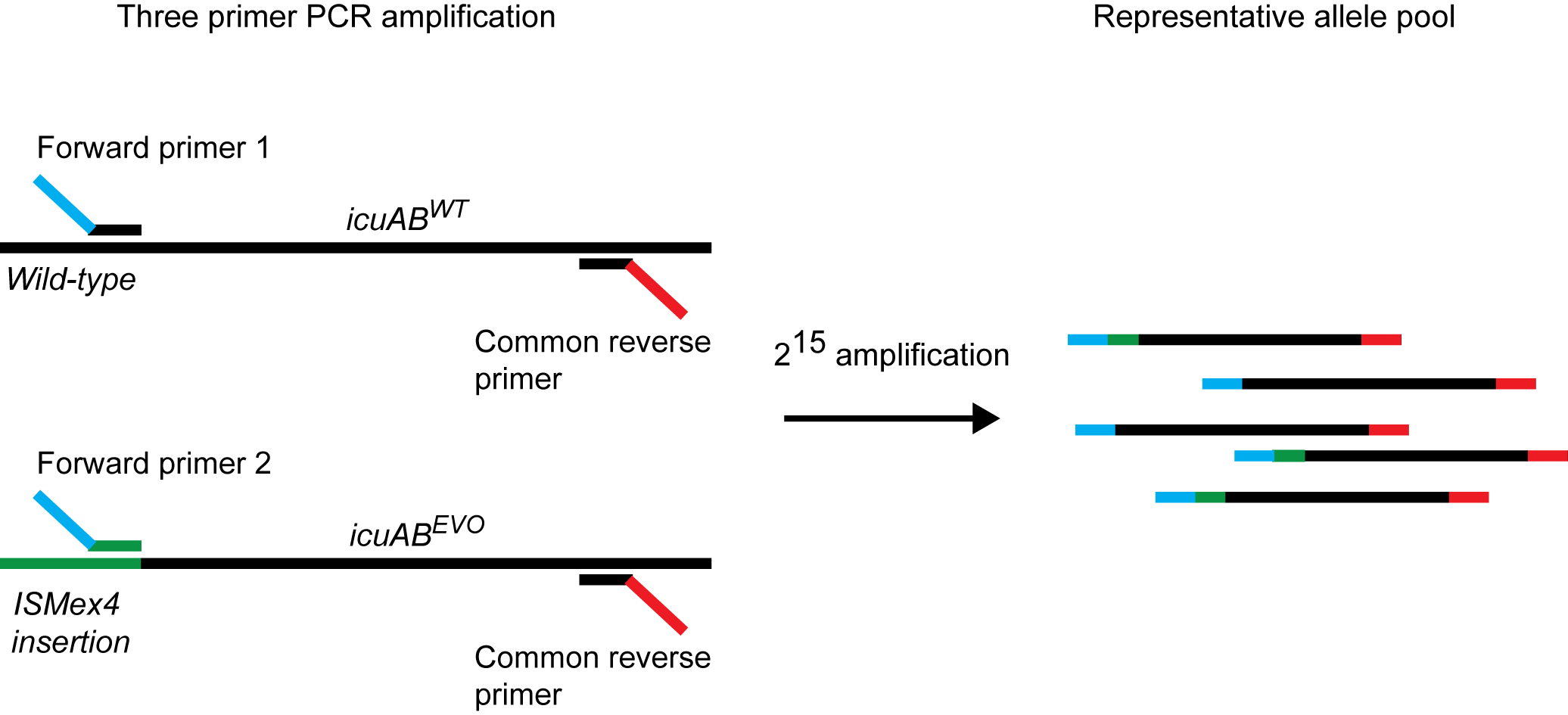

Supplement: Figure S2 — Schematic of the three primer, novel DNA junction implementation of FREQ-Seq. (TIF) [file pone.0047959.s002.tif]

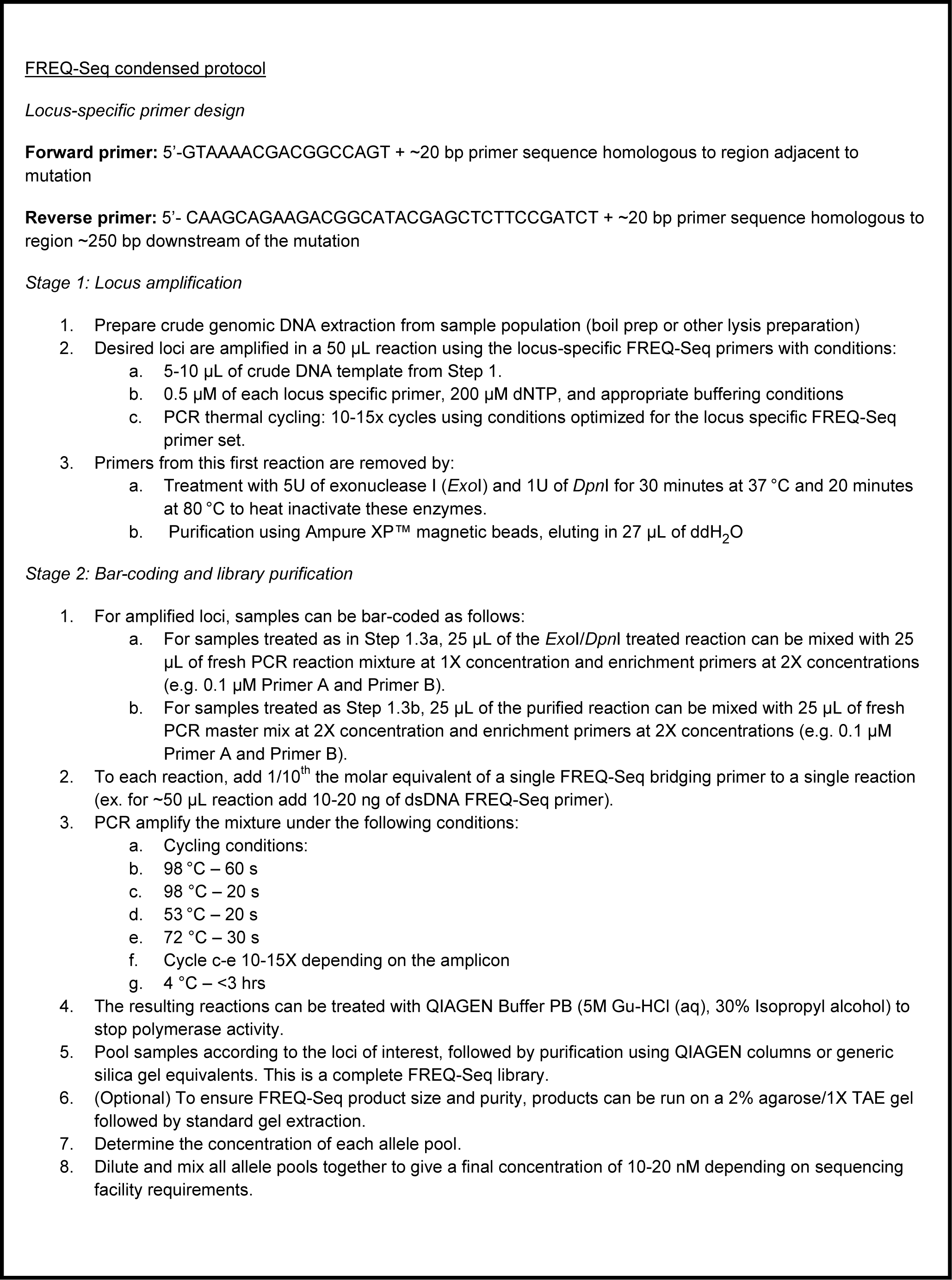

Supplement: Box S1 — Abbreviated FREQ-Seq protocol. (TIF) [file pone.0047959.s003.tif]
