# Supplementary material for: FREQ-Seq: A Rapid, Cost-Effective, Sequencing-Based Method to Determine Allele Frequencies Directly from Mixed Populations
Source: PLoS One. 2012 Oct 31;7(10):e47959. doi: 10.1371/journal.pone.0047959 (PMC3485326; doi:10.1371/journal.pone.0047959)
Supplement: Table S1 — FREQ-Seq primer sequences and allele-specific primers used in this study. (DOCX) [file pone.0047959.s004.docx]

**­­Table S1.** FREQ-Seq primer sequences and allele-specific primers used in this study.

| **Name** | **Sequence** | **Description** |
| --- | --- | --- |
| **FO** | GTAAAACGACGGCCAGT | Forward/sequencing end overhang |
| **RO** | CAAGCAGAAGACGGCATACGAGCTCTTCCGATCT | Single-end read reverse overhang |
| **PRO** | AAGCAGAAGACGGCATACGAGATCGGTCTCGGCATTCCTGCTGAACCGCTCTTCCGATCT | Paired-end read reverse overhang |
| **ABC1** | AATGATACGGCGACCAC | Bar code amplification |
| **ABC2** | ACTGGCCGTCGTTTTAC | Bar code amplification |
| **AF1** | AATGATACGGCGACCAC | FREQ-Seq enrichment |
| **AF2** | CAAGCAGAAGACGGCATAC | FREQ-Seq enrichment |
| **AF3** | **FO**+CAGATCTGAACTTCCCAGCA | *pntAB* forward |
| **AF4** | **RO**+GACCCCAGACCTATGAACTTC | *pntAB* reverse |
| **AF7** | **FO**+ACGCTGCAAGAGTGAACAAC | *gshA* forward |
| **AF8** | **RO**+TGAGATCGATCTGCTGGGTC | *gshA* reverse |
| **AF9** | **FO**+CTAGAGTTCCACGACTTGACAG | *fghA* forward |
| **AF10** | **RO**+CATTTTCATGCGTGCAGGTC | *fghA* reverse |
| **AF11** | **FO**+GATGCTGCGACCGAGATT | *icuAB* forward |
| **AF12** | **FO**+ATCCACTGCCCTCGTGAATA | *icuAB::*ISMex4 forward |
| **AF13** | **RO**+AGTTCCTCCAGCTCAACTGC | *icuAB* reverse |
| **MLBC1** | AATGATACGGCGACCACCGAGATCTACACTCTTTCCCTACACGACGCTCT TCCGATCTGAGAGAGTAAAACGACGGCCAGT | Control bridge adapter |
| **S11** | GACTTCCGGCAAGCTATACG | ISMex25 insertion forward |
| **S12** | CCCGCAAGGAGGGTGAATG | ISMex25 insertion reverse |
