# Supplementary material for: FREQ-Seq: A Rapid, Cost-Effective, Sequencing-Based Method to Determine Allele Frequencies Directly from Mixed Populations
Source: PLoS One. 2012 Oct 31;7(10):e47959. doi: 10.1371/journal.pone.0047959 (PMC3485326; doi:10.1371/journal.pone.0047959)
Supplement: Table S2 — Sequences of the 48 barcodes contained in the FREQ-Seq kit. All sequences are the N bases in the sequence AATGATACGGCGACCACCGAGATCTACACTCTTTCCCTACACGACGCTCTTCCGATCTNNNNNNGTAAAACGACGGCCAGT. (DOCX) [file pone.0047959.s005.docx]

**Table S2.** Sequences of the 48 barcodes contained in the FREQ-Seq kit. All sequences are the N bases in the sequence AATGATACGGCGACCACCGAGATCTACACTCTTTCCCTACACGACGCTCTTCCGATCT**NNNNNN**GTAAAACGACGGCCAGT.

| **Name** | **Sequence** | **Name** | **Sequence** |
| --- | --- | --- | --- |
| BC01 | AGCAAT | BC25 | TGACGA |
| BC02 | CCTGTT | BC26 | CAAATA |
| BC03 | GGGTTT | BC27 | GTTCAG |
| BC04 | GAAGGC | BC28 | CTTCAA |
| BC05 | ATCTCA | BC29 | GTTGGG |
| BC06 | ATGGAT | BC30 | GCTTAG |
| BC07 | ATGTCT | BC31 | TAGCCA |
| BC08 | CGTGAC | BC32 | TAACTT |
| BC09 | TTAGGT | BC33 | CGGATA |
| BC10 | GTGCAT | BC34 | CAGCAG |
| BC11 | AACTTT | BC35 | AAGTAG |
| BC12 | GGATCG | BC36 | GGGACG |
| BC13 | ATAAGG | BC37 | CCGTGG |
| BC14 | ATTGGT | BC38 | ATTGTA |
| BC15 | AGTGAG | BC39 | TTTAGA |
| BC16 | CCCACC | BC40 | CCACGA |
| BC17 | CGATGC | BC41 | TCATGG |
| BC18 | GATAGC | BC42 | GAACCA |
| BC19 | GTCAGA | BC43 | TCCTAA |
| BC20 | TTAAGC | BC44 | CAACGC |
| BC21 | AACCTG | BC45 | AGTGTT |
| BC22 | CTTTGC | BC46 | GGATTA |
| BC23 | TGGAGA | BC47 | TATATA |
| BC24 | AATTGT | BC48 | GTACAA |
