# Supplementary material for: FREQ-Seq: A Rapid, Cost-Effective, Sequencing-Based Method to Determine Allele Frequencies Directly from Mixed Populations
Source: PLoS One. 2012 Oct 31;7(10):e47959. doi: 10.1371/journal.pone.0047959 (PMC3485326; doi:10.1371/journal.pone.0047959)
Supplement: Table S3 — Genotypes of 72 isolates from population F4 at generation 150. An (X) indicates the presence of an evolved allele (EVO) as determined by RFLP or differential PCR amplication. (DOCX) [file pone.0047959.s006.docx]

**Table S3.** Genotypes of 72 isolates from population F4 at generation 150. An (X) indicates the presence of an evolved allele (EVO) as determined by RFLP or differential PCR amplication.

| **F4, G150**  **isolate** | ***trfA*::ISMex25** | ***fghA^EVO^*** | ***gshA^EVO^*** |
| --- | --- | --- | --- |
| **CM3317** |  |  |  |
| **CM3319** |  |  |  |
| **CM3320** |  |  |  |
| **CM3330** |  |  |  |
| **CM3338** |  |  |  |
| **CM3343** |  |  |  |
| **CM3344** |  |  |  |
| **CM3345** |  |  |  |
| **CM3350** |  |  |  |
| **CM3359** |  |  |  |
| **CM3365** |  |  |  |
| **CM3368** |  |  |  |
| **CM3370** |  |  |  |
| **CM3373** |  |  |  |
| **CM3307** |  |  | X |
| **CM3312** |  |  | X |
| **CM3318** |  |  | X |
| **CM3336** |  |  | X |
| **CM3355** |  |  | X |
| **CM3305** |  | X | X |
| **CM3308** |  | X | X |
| **CM3327** |  | X | X |
| **CM3335** |  | X | X |
| **CM3339** |  | X | X |
| **CM3348** |  | X | X |
| **CM3352** |  | X | X |
| **CM3362** |  | X | X |
| **CM3363** |  | X | X |
| **CM3364** |  | X | X |
| **CM3322** | X |  |  |
| **CM3354** | X |  |  |
| **CM3303** | X |  |  |
| **CM3304** | X |  |  |
| **CM3306** | X |  |  |
| **CM3309** | X |  |  |
| **CM3310** | X |  |  |
| **CM3311** | X |  |  |
| **CM3313** | X |  |  |
| **CM3314** | X |  |  |
| **CM3315** | X |  |  |
| **CM3316** | X |  |  |
| **CM3321** | X |  |  |
| **CM3323** | X |  |  |
| **CM3324** | X |  |  |
| **CM3325** | X |  |  |
| **CM3326** | X |  |  |
| **CM3328** | X |  |  |
| **CM3329** | X |  |  |
| **CM3331** | X |  |  |
| **CM3332** | X |  |  |
| **CM3333** | X |  |  |
| **CM3334** | X |  |  |
| **CM3337** | X |  |  |
| **CM3340** | X |  |  |
| **CM3341** | X |  |  |
| **CM3342** | X |  |  |
| **CM3346** | X |  |  |
| **CM3347** | X |  |  |
| **CM3349** | X |  |  |
| **CM3351** | X |  |  |
| **CM3356** | X |  |  |
| **CM3357** | X |  |  |
| **CM3358** | X |  |  |
| **CM3360** | X |  |  |
| **CM3361** | X |  |  |
| **CM3366** | X |  |  |
| **CM3367** | X |  |  |
| **CM3371** | X |  |  |
| **CM3372** | X |  |  |
| **CM3374** | X |  |  |
| **CM3353** | X |  | X |
| **CM3369** | X |  | X |
