# Supplementary material for: FREQ-Seq: A Rapid, Cost-Effective, Sequencing-Based Method to Determine Allele Frequencies Directly from Mixed Populations
Source: PLoS One. 2012 Oct 31;7(10):e47959. doi: 10.1371/journal.pone.0047959 (PMC3485326; doi:10.1371/journal.pone.0047959)
Supplement: Table S4 — Bacterial strains used in this study and their relevant genotypes. (DOCX) [file pone.0047959.s007.docx]

**Table S4.** Bacterial strains used in this study and their relevant genotypes.

| **Strain** |  | | **Relevant genotype or characteristics** | **Reference** |
| --- | --- | --- | --- | --- |
| **NEB10β** | | *E. coli* | *recA1 endA1 rpsL* Φ80(*lacZ*ΔM15) | NEB |
| **CM502** | | *M. extorquens* | *crtI^502^* | [S2] |
| **CM701** | | *M. extorquens* | Δ*mptG* / pCM410 | [23] |
| **CM1145** | | *M. extorquens* | Δ*mptG crtI^502^ gshA^EVO^ pntAB^EVO^* *icuAB^EVO^*/ pCM410.1145 [*fghA^EVO^*] | [23] |
| **CM1175** | | *M. extorquens* | Δ*katA*::[*loxP*-*t_rrnB_*-*P_tacA_*-*mCherry-t_T7_*] | [37] |
| **CM1290** | | *M. extorquens* | Δ*mptG crtI^502^ pntA^EVO^* | [23] |
| **CM1298** | | *M. extorquens* | Δ*mptG crtI^502^ gshA^EVO^* | [23] |
| **CM3137^a^** | | *M. extorquens* | CM1290 *mptG^WT^* | This work |
| **CM3138^a^** | | *M. extorquens* | CM1298 *mptG^WT^* | This work |
| **CM3277** | | *M. extorquens* | CM502 / pCM410.1145 [*fghA^EVO^*] | This work |
| **CM3943** | | *M. extorquens* | CM1175 / pCM410 | This work |

a. The wild-type *mptG* allele was restored into the respective strains using pCM436 following the sucrose counter-selection method described by C.J. Marx [S2]
