# Supplementary material for: FREQ-Seq: A Rapid, Cost-Effective, Sequencing-Based Method to Determine Allele Frequencies Directly from Mixed Populations
Source: PLoS One. 2012 Oct 31;7(10):e47959. doi: 10.1371/journal.pone.0047959 (PMC3485326; doi:10.1371/journal.pone.0047959)
Supplement: Text S1 — (DOCX) [file pone.0047959.s008.docx]

**Supplementary Text**

**Text S1: FREQ-Seq protocol**

Primer design

To implement FREQ-Seq, users must design a single set of oligonucleotide primers for each experimental locus. Using the sequences provided in Table S1, primers for single-end read flow cells are designed as below:

*Sequencing end*

5’-GTAAAACGACGGCCAGT + ~20 bp primer sequence homologous to region adjacent to mutation

*Non-sequencing end*

5’- CAAGCAGAAGACGGCATACGAGCTCTTCCGATCT + ~20 bp primer sequence homologous to region ~250 bp downstream of the mutation

Bridging primer synthesis

Synthesis of the FREQ-Seq bar-coded, bridging primers can be performed in house using a thermal cycler and standard PCR reagents with any polymerase that generates blunt-ended products, such as *Pfu*, DeepVent_R_™ or Phusion™. Example reaction conditions using Phusion™ polymerase are as follows:

DNA template: FREQ-Seq plasmid (~ 1-10 pg)

Oligo primers: ABC1 and ABC2 (0.5 μM each)

Cycling conditions:

98 °C – 60 s

Cycle 30x:

98 °C – 20 s

52 °C – 15 s

72 °C – 5 s

4 °C – <3 hrs or freeze

We have tried three methods for purification of the dsDNA briding primers with success:

1. 2% agarose/1X TAE separation followed by gel extraction using a ZR-96 Zymo gel recovery kit. This provides pure bridge adapter preparations and is compatible with preparing large numbers adapter simultaneously.
2. 10% polyacrylamide/1X TAE separation followed by crush and soak (overnight) extraction, and EtOH precipitation of DNA products. This results in the purest bridge primer preparations, though not typically required.
3. Purification using Ampure XP™ magnetic beads. Best results are achieved if PCR reactions are treated with *Dpn*I to eliminate plasmid contaminants prior to purification. The most rapid cleanup, but results in less pure bridge adapter preparations.

FREQ-Seq library preparation

The FREQ-Seq library preparation requires two stages of PCR amplification. Reactions can be performed with any blunt-end producing DNA polymerase.

*Stage 1: Locus amplification*

1. For microbial populations, cells can be spun down and resuspended in water or other suitable lysis buffer. Following resuspension, cells are boiled at 98-100 °C in a thermal cycler or appropriate water bath. Cell debris can then be removed by centrifugation. The supernatant contains a crude mixture of microbial DNA representative of the original population.
2. Desired loci are amplified in a 50 μL reaction using the locus specific FREQ-Seq primers with conditions:
   1. 5-10 μL of crude DNA template from 1.
   2. 0.5 μM of each primer, 200 μM dNTP, and appropriate buffering conditions
   3. PCR thermal cycling: 10-15X cycles using conditions optimized for the locus specific FREQ-Seq primer set.
3. Primers from this first reaction are removed by:
   1. Treatment with 5U of exonuclease I (*Exo*I) and 1U of *Dpn*I for 30 minutes at 37 °C and 20 minutes at 80 °C to heat inactivate these enzymes. *Dpn*I helps remove methylated chromosomal DNA templates. This reaction can be performed in the PCR reaction buffer. Fastest for large sample numbers.
   2. Purification using Ampure XP™ magnetic beads, eluting in 27 μL of ddH_2_O.

*Stage 2: Bar-coding and library purification*

1. For amplified loci, samples can be bar-coded as follows:
   1. For samples treated as 1.3a (above), 25 μL of the *Exo*I/*Dpn*I treated reaction can be mixed with 25 μL of fresh PCR master mix at 1X concentration and enrichment primers at 2X concentrations (ex. 0.1 μM Primer A and Primer B).
   2. For samples treated as 1.3b (above), 25 μL of the purified reaction can be mixed with 25 μL of fresh PCR master mix at 2X concentration and enrichment primers at 2X concentrations (ex. 0.1 μM Primer A and Primer B).
2. To each reaction, add 1/10^th^ the molar equivalent of a single FREQ-Seq bridging primer to a single reaction (ex. for ~50 μL reaction add 10-20 ng of dsDNA FREQ-Seq primer).
3. PCR amplify the mixture under the following conditions:
   1. Cycling conditions:
   2. 98 °C – 60 s
   3. 98 °C – 20 s
   4. 53 °C – 20 s
   5. 72 °C – 30 s
   6. Cycle c-e 10-15X depending on the amplicon
   7. 4 °C – <3 hrs or freeze
4. The resulting reactions can be treated with QIAGEN Buffer PB (5M Guanidine-HCl (aq), 30% Isopropyl alcohol) to stop polymerase activity.
5. Pool samples according to the loci of interest, followed by purification using QIAGEN columns or generic silica gel equivalents. This is a complete FREQ-Seq library.
6. (Optional) To ensure FREQ-Seq product size and purity, products can be run on a 2% agarose/1X TAE gel followed by standard gel extraction.
7. Determine the concentration of each allele pool.
8. Dilute and mix all allele pools together to give a final concentration of 10-20 nM depending on your sequencing facility requirements.

**Text S2: Description of the FREQout Parsing Steps**

Freq-Out is the companion program to this method which takes as input the FASTQ files from an Illumina run and produces as output the frequency of each type as different barcodes. We briefly described the steps in this process here, but more information and the source code is available at [www.evolvedmicrobe.com/FreqSeq/index.html](http://www.evolvedmicrobe.com/FreqSeq/index.html).

The FREQout Data Parsing Process

1. **Specify Settings:** Users specify the barcodes and allele types they are using in an XML file. FREQout uses the settings in this file to parse the FASTQ files, and for each read either assign it to a specific barcode and allele combination, or discard it if the match is poor or certain quality controls are not passed.
2. **Determine Quality Filters:** The most computationally intense portion of Freq-Out is the task of assigning reads that are not exact matches to known barcode and allele combinations. The program optionally allows users to avoid performing this step for low quality data. This not only allows for faster parsing but also can avoid poor quality matches. Unfortunately, defining low quality data is complicated because there are several variants of the way FASTQ files encode quality scores, although a user typically does not know which format any particular file is in. To circumvent this issue, FREQout instead estimates the distribution of the average quality score for reads in a FASTQ file by parsing a user specified number of reads from the beginning of the file. If the user sets a quality score percentile cut-off, FREQout estimates the average read quality score at this percentile using this empirical distribution, and does not attempt to assign any reads that are below this threshold if they are not an exact match. By default, FREQout will parse the first 5,000 reads to estimate the quality score, and not align any read with an average quality score below the bottom 2% of the quality scores in these initial reads.
3. **Parse the Reads** – FREQout next assigns, in parallel, each read to either a barcode/allele combination, or determines that the read cannot be assigned. For each read it performs the following steps.
   1. **Filters the read based on the presence of the M13f sequence** that should be in every read produced by the FreqSeq method. However, this step is skipped if the user does not require this sequence. If they do (this is the default option), the read is immediately excluded if an exact match is not found unless they also specify the option to allow reads within a Hamming distance of one. If so, the read can still be accepted if it has an M13f sequence that is within a Hamming distance of 1 to the actual sequence.
   2. **Assigns the read to a barcode** – If the read contains an exact match to a barcode it is simply assigned to that group. If not, and if the user has specified the option to allow inexact matches, the read is assigned to a barcode group if the 6 bp barcode sequence is within a Hamming distance of 1 to that barcode (this is the default option provided all barcodes are separated by a Hamming distance over two, which is true for all barcodes considered in this paper).
   3. **Assign the read to an allele type -** If the read contains an exact match to an allele it is assigned to that group. If not, and if the user has set the file to allow for inexact matches the following steps are taken.
      1. Determine if the read passes the quality threshold set in step 2, and also the user specified maximum percentage of “N” basepairs to be considered for inexact alignment.
      2. If the read passes (i), then a list of candidate allelic types is aligned against the sequence to determine if any are significant matches. First all 7 bp K-mers in the read (after trimming the barcode and M13f sequence) are queried against a dictionary of all 7 bp K-mers made from the available allelic types set by the XML file. For each allelic type that matches a K-mer, a Smith-Waterman alignment is done between the type and the sequence from the read. The parameters for the alignment are set in the XML file. If only one allelic type is matched, that allelic type is assigned. If more than two alignments are performed, the allelic type with the highest scoring alignment is assigned, provided that the difference between the highest scoring alignment and the next highest scoring alignment is greater than the Smith-Waterman match score plus the absolute value of the mismatch score (to ensuring the difference is at least equivalent to one SNP in an ungapped alignment).
      3. Update the list of the relevant barcode/allele group with the average quality score for that read.
4. **Output the Results** – FREQout next outputs the results to a comma separated value (csv) file. The output includes the number of reads that were parsed and excluded at each quality step, as well as the number that were assigned as exact or inexact matches and relevant quality scores for each group. Frequencies of each type for each barcode are derived from these quantities.

**Supplemental Methods**

**Allele frequencies determined by restriction fragment length polymorphism.** The FREQ-Seq libraries were first amplified by allele specific primers with M13f and Illumina B overhangs (15 cycles). The PCR products were purified using a QIAGEN PCR purification kit. Purified products were further PCR amplified in a second step using the universal primers (AF1 and AF2) with a small amount (0.02 µM) of barcode primer (MLBC1) (15 cycles). For small indel alleles (*gshA* and *fghA*), we applied reconditioning PCR to reduce the heteroduplex structure of amplified products in each step of PCR (Supplemental Reference 1). These additional PCR amplifications were run for only 2 cycles with excess primers, using the previous 15-cycle PCR product as the template. Endonuclease digestion reactions were carried out with specific enzymes for each allele and purified by QIAGEN MinElute kit. The alleles *pntAB*, *gshA*, and *fghA* were digested with enzymes *Alu*I, *Hha*I, and *Hpy*AV (New England Biolabs), respectively. The purified digests were then run on an Agilent 2100 Bioanalyzer using the DNA1000 kit. The frequency of each allele was estimated using the Bioanalyzer 2100 Expert software.

Supplemental References

1. Thompson, J.R., Marcelino, L.A. and Polz, M.F. (2002) Heteroduplexes in mixed-template amplifications: formation, consequence and elimination by 'reconditioning PCR'. *Nucleic Acids Res*, **30**, 2083-2088.

2. Marx, C.J. (2008) Development of a broad-host-range *sacB*-based vector for unmarked allelic exchange. *BMC Res Notes*, **1**, 1.
